# Comparative assessment of vaccine vectors encoding ten malaria antigens identifies two protective liver-stage candidates

**DOI:** 10.1038/srep11820

**Published:** 2015-07-03

**Authors:** Rhea J. Longley, Ahmed M. Salman, Matthew G. Cottingham, Katie Ewer, Chris J. Janse, Shahid M. Khan, Alexandra J. Spencer, Adrian V. S. Hill

**Affiliations:** 1 The Jenner Institute, University of Oxford, Oxford OX3 7DQ, United Kingdom; 2 Department of Parasitology, Leiden University Medical Center, 2333 ZA Leiden, The Netherlands

## Abstract

The development of an efficacious *Plasmodium falciparum* malaria vaccine remains a top priority for global health. Vaccination with irradiated sporozoites is able to provide complete sterile protection through the action of CD8^+^ T cells at the liver-stage of infection. However, this method is currently unsuitable for large-scale deployment and focus has instead turned to the development of sub-unit vaccines. Sub-unit vaccine efforts have traditionally focused on two well-known pre-erythrocytic antigens, CSP and TRAP, yet thousands of genes are expressed in the liver-stage. We sought to assess the ability of eight alternative *P. falciparum* pre-erythrocytic antigens to induce a high proportion of CD8^+^ T cells. We show that all antigens, when expressed individually in the non-replicating viral vectors ChAd63 and MVA, are capable of inducing an immune response in mice. Furthermore, we also developed chimeric *P. berghei* parasites expressing the cognate *P. falciparum* antigen to enable assessment of efficacy in mice. Our preliminary results indicate that vectors encoding either PfLSA1 or PfLSAP2 are capable of inducing sterile protection dependent on the presence of CD8^+^ T cells. This work has identified two promising *P. falciparum* liver-stage candidate antigens that will now undergo further testing in humans.

Development of a vaccine against the *Plasmodium* parasite, the causative agent of malaria, has proven more difficult than for other pathogens, largely because of its complex life-cycle, its thousands of antigens and its immune evasion mechanisms. The “gold-standard” malaria vaccine (the most effective in human challenge trials) is the administration of irradiated sporozoites^1^, yet despite encouraging developments^2^ this method of vaccination still appears unsuitable for large-scale deployment. Irradiated sporozoites are capable of invading hepatocytes but their development is arrested, providing a repertoire of antigens for the immune system to react against whilst not producing a blood-stage (or symptomatic) infection^3^. Protection by irradiated sporozoites in mice and non-human primates is dependent upon CD8^+^ T cells specific for liver-stage antigens^4^,^5^.

An alternative approach to a malaria vaccine is the development of sub-unit vaccines comprising a particular antigen expressed at one or more stages of the parasite’s life-cycle. The most advanced sub-unit vaccine, RTS,S/AS01, which targets the circumsporozoite protein (CSP) at the pre-erythrocytic stage, may be licensed in the near future but still lacks high levels of durable efficacy^6^. The vaccine is aimed at inducing high titre antibodies to block the sporozoites prior to infection of hepatocytes. The alternative sub-unit vaccination strategy is the induction of high numbers of CD8^+^ T cells to kill infected hepatocytes. The most successful regimen to date has been the use of viral vectors expressing the chosen antigen in a heterologous prime-boost regimen, as for the ME-TRAP vaccine. The ME-TRAP vaccine combines the pre-erythrocytic antigen thrombospondin-related adhesion protein (TRAP) with a multi-epitope string (ME) and is delivered via the viral vectors chimpanzee adenovirus 63 (ChAd63) and modified vaccinia virus Ankara (MVA)^7^. Whilst this vaccine displays moderate levels of efficacy in naïve-adults, it induces exceptionally high CD8^+^ T cell responses. A number of approaches are being assessed with the aim of increasing the efficacy of such sub-unit vaccines, including the use of new adjuvants, different sub-unit vaccination platforms and the use or addition of new antigens.

There is increasing evidence that antigens other than CSP or TRAP may contribute to a protective immune response^8^,^9^,^10^,^11^, and it is likely that multiple antigens will be needed to reach the high levels of efficacy achievable with large doses of irradiated sporozoites. However, only a few antigens have been assessed as sub-unit vaccines partly owing to the difficulty in screening *P. falciparum* vaccines pre-clinically. *P. falciparum* accounts for the majority of the malaria burden in humans, but it does not naturally infect small animals. Therefore, rodent malaria parasite species are routinely used for proof-of-concept studies, yet several newly identified *P. falciparum* antigen candidates do not have orthologs in murine malaria parasite species. Another strategy to study *P. falciparum* immunology and assess malaria vaccines has been the generation of transgenic rodent malaria parasites expressing *P. falciparum* proteins^12^.

In this study, we sought to determine whether eight alternative *P. falciparum* liver-stage antigens could induce strong CD8^+^ T cell responses when delivered using a heterologous ChAd63-MVA prime-boost vaccination regimen. Next, in an effort to determine efficacy of these vaccines, we created ten transgenic *P. berghei* parasites, eight that express these new candidate *P. falciparum* antigens and another two expressing *P. falciparum* CSP or TRAP, enabling a homologous efficacy challenge in mice. Here, we report the successful production of eight vaccines inducing strong CD8^+^ T cell responses and preliminary results demonstrating superior efficacy of ChAd63-MVA prime-boost vaccines encoding one of two antigens, *P. falciparum* (Pf) liver-stage antigen 1 (LSA1) or liver-stage associated protein 2 (LSAP2), when compared to both PfCSP and PfTRAP.

## Results

### Immunogenicity of the candidate vaccines in inbred mice

**Figure 1:**
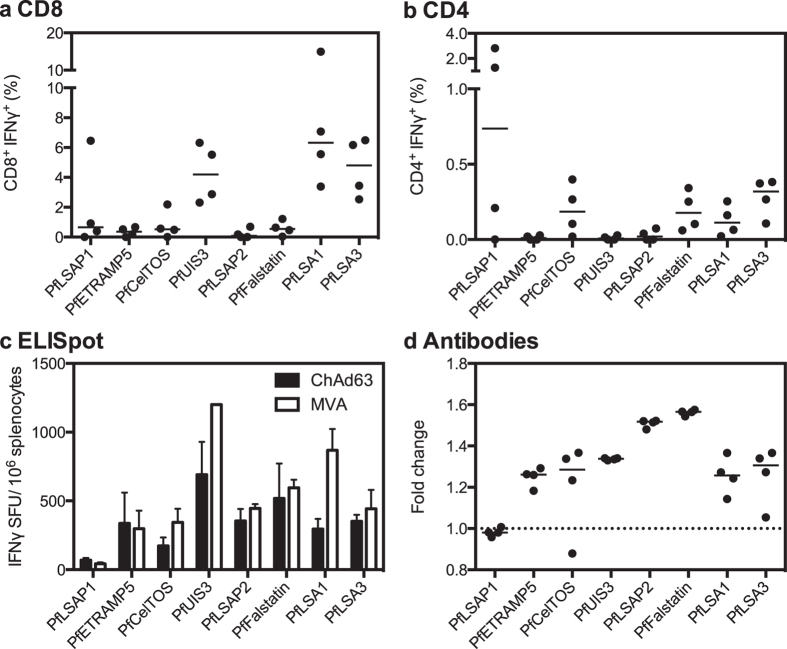
Immunogenicity in BALB/c mice. Panels **a** and **b** show CD8^+^ IFNγ^+^ (**a**) and CD4^+^ IFNγ^+^ (**b**) responses measured in the blood one week post-MVA boost, expressed as the percentage of total CD8^+^ or CD4^+^ cells. Individual data points and the median of four biological replicates are shown. Panel **c** shows total cellular IFNγ responses measured in splenocytes at both two weeks post-prime and two weeks post-boost by *ex vivo* ELISpot. Data are shown as the median IFNγ SFU per million splenocytes with error bars representing the interquartile range of four biological replicates. For the vaccine PfUIS3, a maximum response detectable by ELISpot (due to blackout of the wells) was reached at two weeks post-boost and hence the arbitrary value of 1200 SFU was assigned. Panel **d** shows antibody responses measured in the sera two weeks post-boost. Data are expressed as the fold change from naïve, with individual data points and the median of four biological replicates shown. The dashed line represents no change from naïve (=1).

We first assessed whether the candidate vaccines could induce cellular and humoral immune responses in BALB/c mice using a heterologous ChAd63-MVA prime-boost intramuscular (i.m.) regimen with an eight-week interval (Fig. 1). Specifically, we assessed five previously tested antigens, PfLSA1^13^, liver-stage antigen 3 (PfLSA3)^14^, cell traversal protein for ookinetes and sporozoites (PfCelTOS)^15^, upregulated in sporozoite 3 (PfUIS3) and PfFalstatin^16^, as well as three more recently identified candidates liver-stage associated protein 1 (PfLSAP1), PfLSAP2^17^ and the early transcribed membrane protein 5 (PfETRAMP5)^18^. Candidate antigens were chosen based on three criteria: evidence of being expressed at a high level at the liver-stage; of being targets of a T cell response in natural infection or during irradiated sporozoite immunization; and evidence of protection with vaccine constructs using a rodent parasite ortholog or with *P. falciparum* in non-human primates.

T cell responses to all antigens, predominantly CD8^+^ IFNγ^+^ cells, were detected in the blood one week after MVA boost, with the highest frequencies observed after vaccination with PfLSA1, PfLSA3 and PfUIS3 (medians of 6.3%, 4.8% and 4.2% of CD8^+^ cells producing IFNγ, respectively) (Fig. 1a). CD4^+^ IFNγ^+^ cells were also detected, albeit at a frequency of less than 0.5% of total CD4^+^ cells for most antigens (Fig. 1b). A more sensitive method of detecting cellular responses, *ex vivo* IFNγ enzyme-linked immunosorbent spot (ELISpot) assay, was also used to assess responses in the spleen post-ChAd63 and post-MVA (Fig. 1c). One week after MVA boost the highest immunogenicity levels observed were against the antigens PfUIS3 and PfLSA1 (medians of 1200 and 868 spot forming units (SFU) per million splenocytes, respectively). Induction of humoral immunity was assessed through the luminescence immunoprecipitation system (LIPS)^19^. Detectable antibodies, as determined by the fold change in comparison to naïve mice, were observed against all antigens except PfLSAP1 (Fig. 1d). The greatest magnitude responses were observed for PfFalstatin and PfLSAP2, with a fold change of 1.6 and 1.5, respectively.

**Figure 2:**
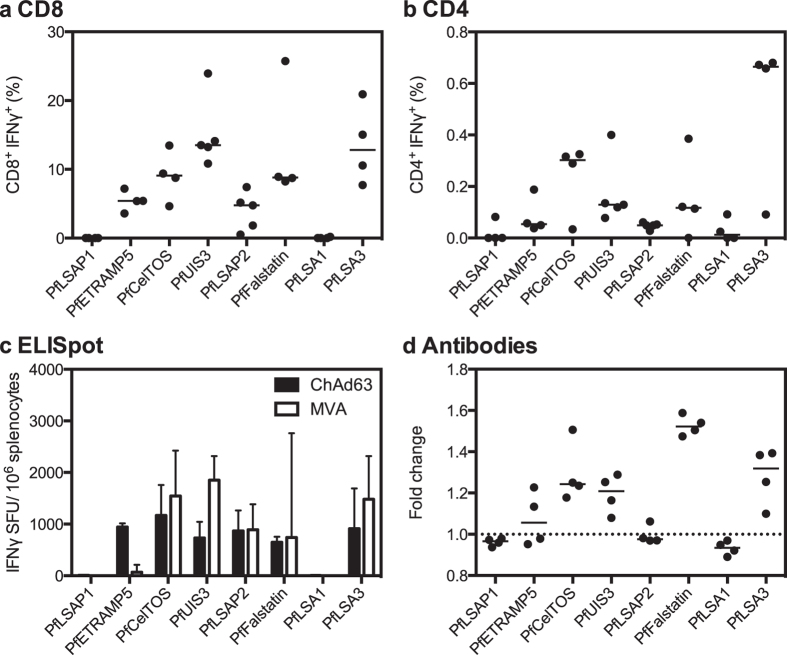
Immunogenicity in C57BL/6 mice. Panels **a** and **b** show CD8^+^ IFNγ^+^ (**a**) and CD4^+^ IFNγ^+^ (**b**) responses measured in the blood one week post-MVA boost, expressed as the percentage of total CD8^+^ or CD4^+^ cells. Individual data points and the median of four or five biological replicates are shown. Panel **c** shows total cellular IFNγ responses measured in splenocytes at both two weeks post-prime and two weeks post-boost by *ex vivo* ELISpot. Data are represented as the median IFNγ SFU per million splenocytes with error bars representing the interquartile range of four to five biological replicates. Panel **d** shows antibody responses measured in the sera two weeks post-boost. Data are expressed as the fold change from naïve, with individual data points and the median of four biological replicates shown. The dashed line represents no change from naïve (=1).

We also assessed whether the candidate vaccines were able to induce immune responses in C57BL/6 mice using the same heterologous ChAd63-MVA prime-boost eight-week interval regimen (Fig. 2). CD8^+^ IFNγ^+^ T cell responses were detected in the blood one week after MVA boost to all antigens except PfLSAP1 and PfLSA1 (Fig. 2a). In general, responses were of higher frequency than seen in BALB/c mice, but with greater levels of variation. Responses of the highest frequency were observed after vaccination with PfUIS3 and PfLSA3 (medians of 13.7% and 12.8% of CD8^+^ producing IFNγ^+^, respectively). As for BALB/c mice, CD4^+^ IFNγ^+^ responses were detected but at a lower frequency (less than 0.8% of total CD4^+^ cells) (Fig. 2b). The splenic IFNγ ELISpot results confirmed an absence of detectable cellular immune responses to PfLSA1 and PfLSAP1 (Fig. 2c). No antibodies were detectable against PfLSAP1, PfLSAP2 and PfLSA1, with a borderline response to PfETRAMP5 (Fig. 2d). The greatest magnitude response was measured for PfFalstatin with a fold change of 1.5, similar to that observed in BALB/c mice.

### Protective efficacy of the candidate vaccines in inbred and outbred mice

We next assessed the protective efficacy of these *P. falciparum* vaccines in inbred and outbred mice, through the use of chimeric *P. berghei* sporozoites expressing the cognate *P. falciparum* antigen. We tested the efficacy of our vaccines in BALB/c mice because a detectable cellular immune response was generated to all antigens and because these responses were more consistent than in C57BL/6 mice. We also conducted the same experiments in an outbred strain of mice, CD-1, in order to limit the effect of MHC restriction and immunodominance that can be observed in inbred strains of mice. Following an eight-week interval i.m. vaccination regimen, mice were challenged eight days after MVA boost, which corresponds to the approximate peak of CD8^+^ T cell immunogenicity^20^, with 1000 chimeric sporozoites injected intravenously (i.v.) to focus the challenge to a liver-stage infection. PfCSP and PfTRAP were used as control antigens. For all challenge experiments, statistically significant efficacy (sterile protection or delay) was assessed using the Log-Rank (Mantel-Cox) Test.

Vaccination with PfTRAP did not induce a statistically significant level of sterile protection in the chimeric parasite model in BALB/c mice, but did protect 3/10 (30%) CD-1 mice (Table 1). Vaccination with PfCSP protected 3/8 BALB/c mice (37.5%) and 3/9 (33.3%) CD-1 mice. The remaining mice had a significant delay in the time to 1% parasitaemia, with a median delay of 1.47 (p = 0.0008) and 0.48 days (p = 0.001) in BALB/c and CD-1 mice respectively. In comparison, vaccination with PfLSA1 protected 7/8 (87.5%) of both BALB/c and CD-1 mice, a statistically significantly higher proportion than of unvaccinated mice (p < 0.0001). Similarly, vaccination with PfLSAP2 protected 7/8 (87.5%) BALB/c mice and 7/10 (70%) CD-1 mice (p < 0.0001 and p = 0.0009, respectively).

Vaccination with PfUIS3 induced sterile protection in 1/8 BALB/c mice (12.5%), and a significant delay in the time to 1% parasitaemia compared to naïve controls was also observed (1.42 days, p = 0.004, overall p = 0.0001), similar to that observed after PfCSP vaccination. However, PfUIS3 vaccination was unable to confer protection in CD-1 mice despite a non-significant trend towards delayed parasitaemia (median delay of 1.10 days compared with naïve controls). PfFalstatin was also able to induce a statistically significant but small delay in the time to 1% parasitaemia in both BALB/c and CD-1 mice (median delay of 0.59 days, p = 0.007, and 0.97 days, p < 0.0001, respectively). PfLSA3 was able to induce a statistically significant but small delay in the time to 1% parasitaemia in BALB/c mice (median 0.35 days, p = 0.03), but not in CD-1 mice. PfCelTOS, PfLSAP1 or PfETRAMP5 vaccination was unable to provide sterile protection or a delay in the time to 1% parasitaemia in BALB/c or CD-1 mice. Kaplan-Meier survival curves are provided in the Supplementary Information (Supplementary Figs S1-2).

### Mechanism of protective efficacy in BALB/c mice

**Figure 3:**
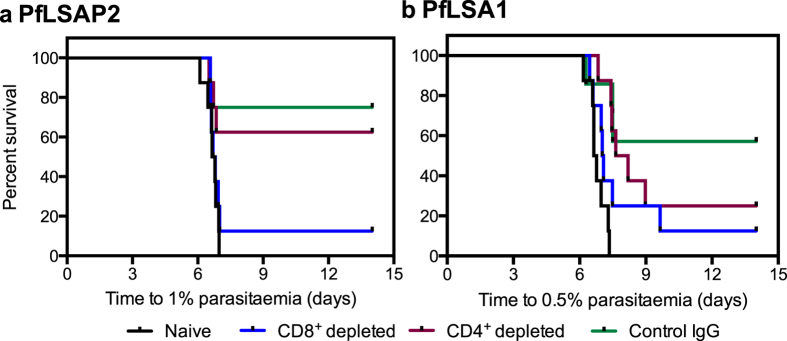
CD8^+^ T cells are required for protective efficacy. BALB/c mice (n = 7–8 per group) were injected with the appropriate monoclonal antibody to deplete CD4^+^ or CD8^+^ T cells, or with an unrelated IgG control, and challenged with 1000 chimeric parasites i.v. ten days after ChAd63-MVA vaccination. Naïve mice acted as another control. The Kaplan-Meier curves illustrate the time to 0.5 or 1% parasitaemia, and the Log-Rank (Mantel-Cox) Test was used to compare groups of mice. For PfLSAP2 (panel **a**): CD8^+^ depleted vs naïve, not significant (NS); CD8^+^ depleted vs control IgG, p = 0.03; CD4^+^ depleted vs naïve, p = 0.01; and CD4^+^ depleted vs control IgG, NS. For PfLSA1 (panel **b**): CD8^+^ depleted vs naïve, NS; CD8^+^ depleted vs control IgG, NS; CD4^+^ depleted vs naïve, p = 0.0003; CD4^+^ depleted vs control IgG, NS.

*P. falciparum* antigen expression in the chimeric parasites was under the control of the *P. berghei* upregulated in sporozoites 4 (*UIS4*) promoter, leading to expression both in sporozoites and during the liver stage^21^. Whilst use of the i.v. route of challenge permitted focus on the liver-stage of infection, protection could still have been mediated via antibody inhibition of sporozoite invasion and/or cell-mediated killing of infected hepatocytes. We depleted BALB/c mice of CD8^+^ and CD4^+^ T cells before challenge to determine the reliance on T cells and the relative contribution of each sub-set to protection (Fig. 3), using PfLSAP2 and PfLSA1 as model antigens given their superior efficacy against chimeric parasites.

For PfLSAP2 vaccination, protection was dependent on the presence of CD8^+^ T cells (Fig. 3a). CD8^+^ depletion reduced sterile protection from 6/8 (75%) in the IgG control group to 1/8 (12.5%) (p = 0.03) and there was no statistically significant difference between the CD8^+^ depleted group and naïve mice. CD4^+^ T cells did not appear to play a protective role following vaccination with PfLSAP2, since no significant difference compared to the IgG control group was observed, with 5/8 (62.5%) mice remaining sterilely protected. Furthermore, there was a statistically significant difference between the CD4^+^ depleted group and naïve mice (p = 0.01).

For PfLSA1 vaccination, the contribution of CD8^+^ T cells was not as clear as for PfLSAP2 (Fig. 3b). CD8^+^ depletion reduced sterile protection from 4/7 (57.1%) in the IgG control group to 1/8 (12.5%), but did not reach statistical significance (p = 0.07). 2/8 (25%) mice were sterilely protected after CD4^+^ depletion, a statistically greater level than compared to the naïve control group (p = 0.0003), suggesting these mice are still afforded with some protection.

**Figure 4:**
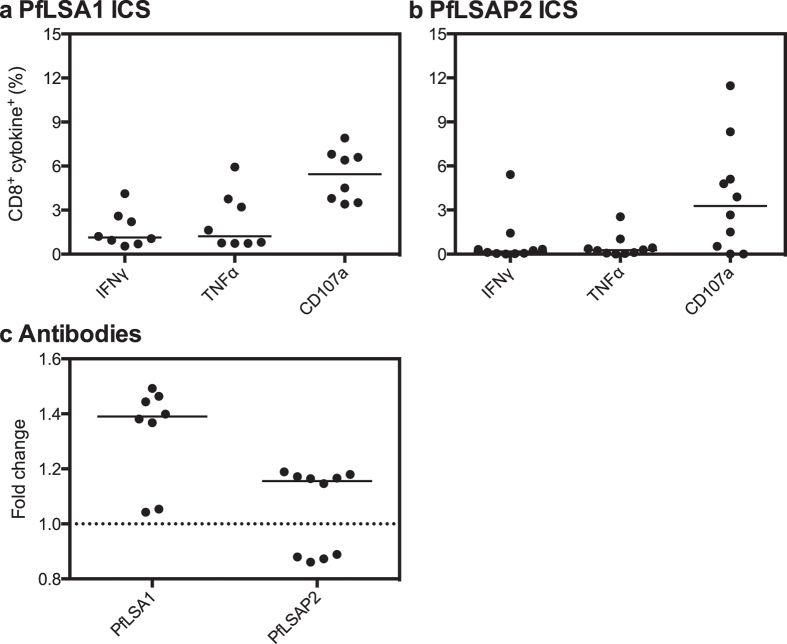
Immunogenicity of PfLSAP2 and PfLSA1 in outbred mice. Panels **a** and **b** show CD8^+^ IFNγ^+^, TNFα^+^ and CD107a^+^ responses measured in (**a**) PfLSA1 and (**b**) PfLSAP2 vaccinated CD-1 mice one week post-MVA boost, expressed as the percentage of total CD8^+^ cells. Individual data points and the median of eight to ten biological replicates are shown. Panel **c** shows the specific antibody response measured in CD-1 mice one week post-MVA boost. Data are expressed as the fold change from naïve, with individual data points and the median of eight to ten biological replicates shown. The dashed line represents no change from naïve (=1).

Whilst we did not conduct depletion experiments in CD-1 mice, we determined that PfLSA1 vaccination was immunogenic in CD-1 mice with a median CD8^+^ IFNγ^+^ response of 1.1% of total CD8^+^ cells, CD8^+^ TNFα^+^ of 1.2% and CD8^+^ CD107a^+^ of 5.5% (Fig. 4a). We also observed a detectable antibody response in 6/8 (75%) CD-1 mice (Fig. 4c). PfLSAP2 was able to induce a low cellular response in CD-1 mice (Fig. 4b). 6/10 PfLSAP2-vaccinated CD-1 mice exhibited a detectable level of antibodies (Fig. 4c), but there was no difference between protected and unprotected mice.

### Generation of antigen-specific CD8^+^ T cells in the liver

**Figure 5:**
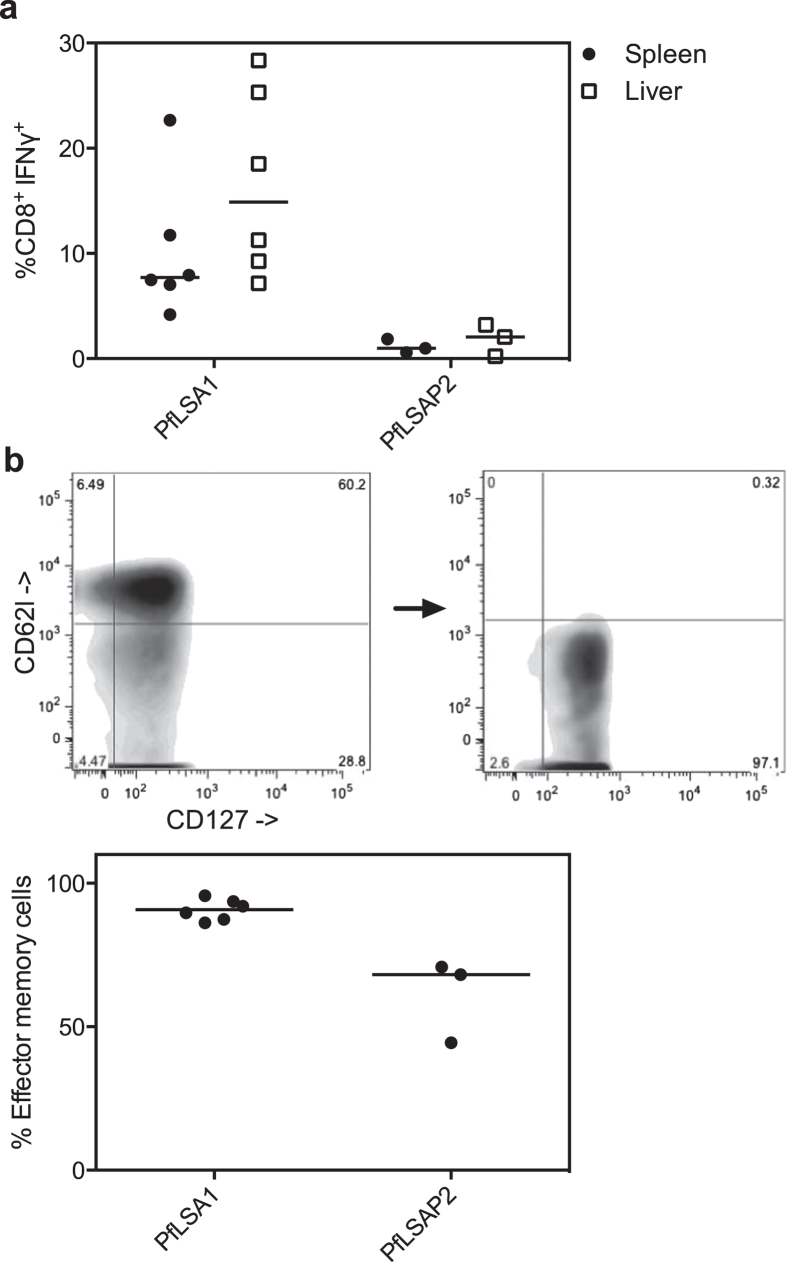
Effector memory CD8^+^ T cells in the liver. Panel **a** shows the CD8^+^ IFNγ^+^ responses measured in the spleen and the liver two weeks post-MVA boost for both PfLSA1 and PfLSAP2 vaccinated BALB/c mice, expressed as the percentage of total CD8^+^ cells. Individual data points and the median of three to six biological replicates are shown. Liver mononuclear cells were also stained with the markers CD62l and CD127 to calculate the percentage of effector memory (CD62l^−^ CD127^+^) and central memory (CD62l^+^ CD127^+^) subsets. The gating strategy used to identify the populations is shown in panel **b**; gating was first performed on CD8^+^ cells, where a clear central memory population can be seen (CD62l^+^ CD127^+^). Antigen-specific cells were then selected based on secretion of IFNγ and a representative population is shown. The graph shows the percentage effector memory population in the liver.

As protection in BALB/c mice was highly reliant on the presence of CD8^+^ T cells, we next determined whether a cellular response was detectable in the liver. Using PfLSAP2 and PfLSA1 as our model antigens, we found that both were capable of inducing an antigen-specific CD8^+^ IFNγ^+^ response in the liver of equal magnitude to that seen in the spleen (Fig. 5a). Of the antigen-specific CD8^+^ T cells present in the liver, the majority expressed markers of the effector memory subtype (CD62l^−^, CD127^+^ 22,23) (Fig. 5b).

## Discussion

The pre-erythrocytic sub-unit vaccines currently undergoing clinical assessment are based on only two antigens, namely PfCSP and PfTRAP. Our results demonstrate that other antigen candidates are also capable of inducing strong CD8^+^ T cell responses and may be better targets for a liver-stage malaria vaccine. Our work highlights the capacity of viral vectored vaccines in general to induce strong cellular immunogenicity, and we consistently found that the antigens PfLSA1, PfLSA3 and PfUIS3 were capable of inducing responses of the highest magnitudes. Approximately twice the frequency of antigen-specific cells was observed after ChAd63-MVA vaccination than previously observed by vaccination with PfCelTOS protein^15^, PfLSA1 protein^24^ or PfLSA3 DNA^14^; only DNA vaccination with *in vivo* electroporation has been shown to be similarly immunogenic^25^. In the current study, responses predominantly comprised CD8^+^ T cells secreting IFNγ, TNFα or with upregulated expression of the cytotoxic marker CD107a.

We chose to use chimeric *P. berghei* parasites expressing the cognate *P. falciparum* antigen in order to assess efficacy in a murine model. We found that vaccines expressing either PfLSA1 or PfLSAP2 were substantially and significantly more effective than both PfCSP and PfTRAP when delivered using the same ChAd63-MVA prime-boost regimen. Vaccination with PfLSA1 or PfLSAP2 sterilely protected 87.5% of BALB/c mice, compared with 37.5% efficacy for PfCSP and 0% efficacy for PfTRAP. Vaccination with PfLSA1 or PfLSAP2 also provided greater than 70% sterile protection in outbred mice, a level of efficacy not previously achieved to our knowledge, and again, greater than that induced by PfCSP (33%) or PfTRAP (30%). Vaccination with PfUIS3, PfFalstatin and PfLSA3 in BALB/c mice provided a degree of protection, manifest largely as a delay in the time to patent parasitaemia, consistent with previous work using murine *Plasmodium* challenges^14^,^16^. Protection was also induced at a low level for PfFalstatin in outbred mice. Surprisingly, no protection was observed after vaccination with PfCelTOS despite previous reports of cross-species protection in murine models^15^.

Neither PfLSA1 nor PfLSAP2 have a known ortholog in a murine parasite species, and hence the classical method of pre-clinical testing using rodent parasite orthologs of *P. falciparum* antigens as vaccine candidates would not have been able to identify these as protective, highlighting the value of the chimeric parasite system described here. There are, however, a number of limitations with this model and hence we consider our results as preliminary. First, despite the use of chimeric parasites it is still a murine model and encounters issues of small numbers of MHC restricted epitopes and marked immunodominance, which is less prominent in human populations^26^. Outbred mice can be used to possibly reflect more accurately what may be seen in a human study and, importantly, efficacy with PfLSA1 and PfLSAP2 was here maintained in CD-1 mice. However, it is possible that some of the candidate antigens that were poorly immunogenic and not protective in outbred mice may still be immunogenic in human populations. Second, the *P. falciparum* antigen is under control of the *P. berghei UIS4* promoter. Whilst this places all antigens on an even level, in terms of expression, it will be important to compare efficacy when expression levels are matched as far as possible to those observed with the native *P. falciparum* parasite, perhaps using replacements of *P. berghei* genes with their *P. falciparum* orthologs. However, this approach is not applicable to the most protective antigens identified, which have no *P. berghei* orthologs. Despite these limitations, the identification of several antigens that could provide greater efficacy than PfCSP or PfTRAP indicates that further antigens warrant assessment in the same system. This study is also the first report of PfLSAP2 as an antigenic target in a malaria vaccine and hence the current findings should encourage further research into its functional role.

The use of a murine model to assess efficacy also allowed us to determine the importance of the CD8^+^ T cells our vaccines induced. We utilized both PfLSA1 and PfLSAP2 as model antigens to further explore the mechanisms of protection given their superior efficacy against chimeric parasites. The depletion experiments highlighted a strong dependence on CD8^+^ T cells for protection after vaccination with both PfLSA1 and PfLSAP2. Both these antigens are likely located in the parasitophorous vacuole membrane (PVM)^17^,^27^, and antigens that reach the PVM or host cell cytoplasm may have epitopes that are better presented on the hepatocyte surface than non-PVM antigens^28^,^29^. PfLSA1 and PfLSAP2 may in this way provide good targets for a cell-mediated immune response. It was also of interest to determine whether these vaccines induced an antigen-specific response in the liver, given that liver-resident CD8^+^ T cells would reduce the time taken to mount an effective immune response. Moreover, it is known that the liver is an immunological organ that is capable of presenting antigen to naïve T cells^30^, and previous studies have demonstrated the presence of liver-resident T cells following vaccination with various malaria constructs^23^,^31^,^32^,^33^,^34^. We similarly found evidence of antigen-specific CD8^+^ cells in the livers of vaccinated mice, largely of the effector memory sub-type with similar frequencies to previously reported results^23^,^35^.

Whilst our efficacy results are preliminary, this work provides strong support for the progression of ChAd63-MVA PfLSA1 to clinical assessment. The level of antigen-specific CD8^+^ T cells induced in mice was of a moderate frequency, and similar levels should be achievable in humans with viral vectors, based on previous studies^7^. PfLSA1 is known to be well conserved amongst *P. falciparum* isolates^36^,^37^ and cellular responses to PfLSA1 have been associated with protection in studies of naturally acquired immunity and in volunteers vaccinated with irradiated sporozoites^38^,^39^,^40^,^41^,^42^,^43^,^44^. Furthermore, PfLSA1 is essential for late liver-stage development^45^, likely involved in the transition from the liver-stage to the blood-stage, as it is expressed abundantly in the PV as flocculent material surrounding merozoites^36^. Whilst a clinical trial of recombinant protein PfLSA1 provided no protection against sporozoite challenge^13^, this was most likely due to the absence of induced CD8^+^ T cells. If the protective effects of ChAd63-MVA PfLSA1 are confirmed in efficacy trials in non-immune adults, this could pave the way for a new generation of malaria vaccines incorporating multiple protective antigens that may be identified using chimeric parasites.

## Materials and Methods

### Vaccine design and generation

All antigen constructs were based on the 3D7 *P. falciparum* sequence from PlasmoDB (http://plasmodb.org/) (see Supplementary Table S1 for accession numbers, size and modifications). The sequences were analysed using the SignalP 3.0 and TMHMM Servers from the Center for Biological Sequence Analysis (http://www.cbs.dtu.dk/services/) to predict subcellular localization and membrane topology. Modifications to the antigen sequence were as follows: the tissue plasminogen activator (tPA) leader sequence (GenBank Accession K03021) was added to the N-terminus of the encoded protein to enhance antigen secretion, expression and immunogenicity^46^. This sequence was not added for PfLSA3, PfLSAP1 and PfUIS3 because these antigens contain two predicted transmembrane domains and so are likely to be anchored in the membrane, based on their predicted subcellular location and membrane topology. The repetitive regions of PfLSA1 were deleted to leave only two copies of the 43 degenerate seventeen amino acid repeats (one of the most and one of the least representative), whilst repetitive regions in PfLSA3 were deleted to leave only one copy.

Coding sequences were synthesized by GeneArt (Life Technologies, USA), with a number of further modifications requested. The sequence was preceded by the Kozak sequence to aid translation in mammalian cells and the *Kpn*1 restriction enzyme site for cloning into the viral vectors. At the 3′ DNA appendix, a STOP codon and the *Not*1 restriction enzyme site were added. No sequences contained the *Vaccinia* virus early gene transcription termination signal 5′- TTTTTNT -3′. All sequences were also optimized to mammalian codon usage bias for expression in human cells.

To generate recombinant adenoviruses, the antigen constructs above were sub-cloned into an entry vector comprising a modified human cytomegalovirus immediate-early promoter with two tetracycline repressor-operator complexes. The modified entry vectors were directionally inserted into the E1 and E3 deleted adenoviral genome at the E1 locus by site-specific recombination. The recombinant adenoviral plasmids were transfected into T-REx™ 293 cells and the adenoviruses purified and titred as previously described^47^. To generate recombinant MVA, the antigen constructs were cloned into a shuttle plasmid with the antigen under control of the p7.5 promoter, to enable markerless insertion at the thymidine kinase locus by recombination and transient-dominant selection in infected cells, as previously described^48^. Recombinant viruses were purified by flow sorting and plaque-picking, and titred as previously described^48^. The identity and purity of the viruses was determined by PCR. Primer sequences are available upon request.

Mice were immunized i.m. into the musculus tibialis with a total volume of 50 μl vaccine administered in endotoxin free D-PBS, with a dose of 1 × 10^8^ infectious units (ifu) for ChAd63 and 1 × 10^7^ plaque forming units (pfu) for MVA in BALB/c and 1x10^6^ pfu MVA in C57BL/6.

### Experimental animals

For experiments undertaken at Oxford University, female BALB/c, C57BL/6 and CD-1 mice, of at least six weeks of age, were purchased from Harlan, UK. All animal work was conducted in accordance with the UK Animals (Scientific Procedures) Act 1986 and approved by the University of Oxford Animal Care and Ethical Review Committee for use under Project License PPL 30/2414 or 30/2889. For the generation of chimeric parasites performed at the Leiden University Medical Centre, female Swiss OF1 mice of six weeks of age were purchased from Charles River Laboratories. All animal work was approved by the Animal Experiments Committee of the Leiden University Medical Centre (DEC 12042). The Dutch Experiments on Animals Act was established under European guidelines (EU directive number 86/609/EEC regarding the Protection of Animals used for Experimental and Other Scientific Purposes).

### Generation of chimeric parasites

Chimeric *P. berghei* parasites containing the *P. falciparum* gene of interest at the neutral *230p* locus were generated following the ‘gene insertion/marker out’ (GIMO) technology as previously described^49^, using the standard GIMO DNA construct pL0043 (see Supplementary Fig. S3). This construct contains 5′ and 3′ targeting sequences for the *230p* locus as well as a multiple-cloning site for integration of transgene-expression cassettes. These constructs integrate by double crossover homologous recombination and replace the positive-negative selectable marker (SM) (human *dihydrofolate* reductase:: yeast *cytosine deaminase* and *uridyl phosphoribosyl transferase* (hdhfr::y*fcu*)) cassette with the transgene-expression cassette. The expression cassette contained the transgene flanked by the 5′ and 3′ promoter and transcription terminator sequences of *P. berghei UIS4*, which were amplified from *P. berghei* ANKA wild-type (WT) genomic DNA^50^, to attempt to standardize expression of all ten transgenes.

The coding sequence of the various *P. falciparum* genes were PCR amplified from *P. falciparum* genomic DNA^51^ (primer sequences are available upon request), apart from LSA1 and LSA3. Due to the large size of these open reading frames the coding sequence was amplified from plasmids used in the generation of the vaccine constructs (and hence codon optimized for expression in mammalian cells). In addition, a reporter-cassette containing GFP::luciferase^52^, driven by the constitutive *P. berghei* elongation factor 1 alpha (ef1α) promoter, was also cloned into each transgene construct. The coding sequence and promoter region of all constructs was confirmed by sequencing.

Linearized constructs were introduced into the GIMO parasites using standard methods of GIMO-transfection^49^. Transfected parasites were selected in mice through addition of 5-fluorocytosine (5-FC) in drinking water^53^, resulting in negative selection of parasites where the SM in the *230p* locus was replaced by the expression/reporter-cassette. All selected chimeric parasites were cloned by limiting dilution^54^ with correct integration confirmed by diagnostic PCR analysis on genomic DNA and Southern analysis of pulsed field gel separated chromosomes, as previously described^50^. Primer sequences are available upon request. Genotype analyses of clones of the ten chimeric lines generated confirmed correct integration of the *P. falciparum* coding sequence into the *P. berghei* genome (see Supplementary Fig. S3).

### Phenotypic analysis of chimeric parasites

Liver-stage expression of the antigen-specific chimeric lines was confirmed indirectly by *in vivo* imaging 44 hours post-challenge in naïve BALB/c mice (see Supplementary Fig. S4), using the IVIS 200 imager, as previously described^55^. Expression of the *P. falciparum* antigens in the chimeric parasites was analysed by immunofluorescence assay (see Supplementary Fig. S5). Chimeric sporozoites were loaded onto glass slides and fixed with 4% paraformaldehyde. The slides were then blocked with 10% FCS and 1% BSA in PBS before the addition of either monoclonal antibodies to *P. berghei* CSP (3D11) or *P. falciparum* CSP (2A10) (MR4, USA), or serum from vaccinated mice. Bound IgG was detected with goat anti-mouse IgG-Alexa Fluor 488 (Life Technologies) and nuclear DNA stained with 2% Hoechst-33342. Slides were mounted with Fluorescence Mounting Medium (Dako, Denmark) and viewed under a Leica DMI-300B microscope.

All transgenic parasites were deemed comparable to wild-type *P. berghei* in terms of infectivity to BALB/c mice (see Supplementary Fig. S6), with two exceptions: the PfUIS3 chimeric was slightly more potent than wild-type (median 5.3 days time to 1% parasitaemia compared to 6.0 days) (p < 0.0001, Mann-Whitney test), and the PfLSA3 chimeric was slightly less potent (median 6.9 days time to 1% parasitaemia compared to 6.0 days) (p = 0.02, Mann-Whitney test). All chimeric parasites were comparable to wild-type *P. berghei* in CD-1 mice, with two exceptions: the PfCSP and PfLSA3 chimerics were slightly less potent than wild-type infection (median time to 1% parasitaemia of 6.35 days in wild-type, compared to 6.77 days for the PfCSP chimeric and 6.96 days for PfLSA3) (p = 0.009 and p = 0.0004, respectively, Mann-Whitney test) (Fig. S7).

### ICS assay

For intracellular cytokine staining (ICS), blood was first lysed with ammonium-chloride-potassium (ACK) lysis buffer to isolate the peripheral blood mononuclear cells followed by stimulation for six hours with a final concentration of 5 μg/ml of the appropriate peptide pool, 1 μg/ml GolgiPlug™ (BD Biosciences, UK), and anti-mouse CD107a-PE. Peptide pools encompassed synthetic 20mers overlapping by ten amino acids (the exception being PfCelTOS, where the pool encompassed synthetic 15mers overlapping by ten amino acids) (peptides synthesized by Neo Group Inc., USA, Mimotopes, UK or Thermo Fisher Scientific, USA). Cells were subsequently surface stained with anti-mouse CD16/32, anti-mouse CD4-eFluor® 450 and anti-mouse CD8α-PerCPCy5.5, whilst intracellular staining was performed using anti-mouse TNFα-FITC, anti-mouse IL-2-PeCy7 and anti-mouse IFNγ-APC. Data were acquired using a LSRII flow cytometer (BD Biosciences) and analysed using FlowJo (Tree Star Inc.). All antibodies were purchased from BD Biosciences or eBioscience, UK.

### Isolation and staining of liver mononuclear cells

Liver mononuclear cells were isolated from sacrificed mice by flushing the circulating blood from the liver with D-PBS, *in situ.* The liver was then dissected and mashed through a 70 μm cell strainer, followed by centrifugation at 480xg for seven minutes. The cell pellet was then resuspended in 10ml of 33% isotonic percoll solution, followed by centrifugation at 693xg for twelve minutes without break. The resulting upper layers were carefully removed and the cell pellet resuspended in ACK lysis buffer to remove any contaminating red blood cells, followed by stimulation for six hours with a final concentration of 5 μg/ml of the appropriate peptide pool, 1 μg/ml GolgiPlug™ and anti-mouse CD107a-PE.

To stain for memory cell markers, the first layer compromised anti-mouse CD16/32, anti-mouse CD8α-PerCPCy5.5, anti-mouse CD4-eFluor® 650, anti-mouse CD62l-PeCy7, anti-mouse CD127-APCeFluor® 780 and LIVE/DEAD® Aqua (Invitrogen, UK). The second layer compromised anti-mouse TNFα-FITC and anti-mouse IFNγ-eFluor® 450. All other steps were identical as for the standard ICS detailed above.

### *Ex vivo* IFNγ ELISpot assay

Splenocytes were treated with ACK lysis buffer followed by stimulation for 18-20 hours with a final concentration of 1 μg/ml of the appropriate peptide pool in MAIP ELISpot plates (Mabtech, Sweden). ELISpots were performed as previously described using coating and detecting antibodies from MabTech^55^. Spots were enumerated using an ELISpot plate counter (AID, Germany) and expressed as the number of spot forming units per million splenocytes, after background subtraction from wells containing cells, media and no peptide.

### LIPS assay

Antibody responses were quantified using the LIPS assay as previously described^19^. Each antigen was expressed in cell culture as an antigen-*Renilla* luciferase (rluc) fusion protein and the cell lysate was harvested. The LIPS assay was initiated by incubation of 1/100 sera with 1 × 10^7^ light units antigen-rluc lysate for one hour in a 96-well V bottom plate on a rotary shaker. The mixture was then transferred to 96-well filter MultiScreen HTS plates (Millipore, USA) containing a 30% solution of protein A/G beads (Fisher Scientific, USA) to capture the antibody during a one-hour incubation period. After washing, antibody-bound antigen-rluc was measured by the addition of the coelenterazine substrate (Promega, USA) and the light emitted measured using a luminometer (Thermo Scientific Varioskan® Flash). The background luminescence was defined as two times the standard deviation plus the average of six naïve serum replicates.

### Efficacy studies

To determine the efficacy of the liver-stage vaccines, chimeric *P. berghei* infected *A. stephensi* mosquitoes were dissected 21 days post-feed to isolate salivary gland sporozoites. 1000 sporozoites were injected i.v. into the tail vein eight days following the final vaccination, corresponding to the peak of cellular immunogenicity. Mice were monitored from four days post-infection by thin film blood smears stained with Giemsa and assessed by light microscopy for the presence of blood-stage parasites. Once positive blood-films had been confirmed on three consecutive days, or mice had reached the end-point of fourteen days parasite free (sterilely protected), mice were culled. The time to 0.5 or 1% parasitaemia was subsequently calculated using linear regression based on the three consecutive thin blood films, dependent on the spread of data collected.

### CD8^+^ and CD4^+^ T cell depletions

Subsets of T cells were depleted using the monoclonal antibodies anti-CD4 clone GK1.5 (rat IgG2a) or anti-CD8 clone 2.43 (rat IgG2a) purified using protein G affinity chromatography from hybridoma culture supernatants. IgG from normal rat serum was purchased (Sigma Aldrich) and purified using the same method. 100 μg of depleting antibody injected intraperitoneal on days −2, −1 and 0 (with respect to challenge on day 0) successfully depleted 100% of either cell population as determined by flow cytometry on day 4.

### Statistical analysis

Prism version 5 (Graphpad, USA) was used for all analyses. LIPS data were log_10_ transformed prior to analysis. Survival in challenge experiments is presented using Kaplan-Meier curves and significance tested using the Log-Rank (Mantel-Cox) Test. For the median delay, statistical significance was assessed after the removal of uninfected mice (sterile protection). The significance threshold was 0.05.

## Additional Information

**How to cite this article**: Longley, R. J. *et al.* Comparative assessment of vaccine vectors encoding ten malaria antigens identifies two protective liver-stage candidates. *Sci. Rep.* **5**, 11820; doi: 10.1038/srep11820 (2015).

## Supplementary Material

Supplementary Information

## Figures and Tables

**Table 1:** Sterile protection and median delay induced by ChAd63-MVA *P. falciparum* vaccines in inbred and outbred mice.

Vaccine	BALB/c	Median delay^b^	CD-1	Median delay^b^
	Protection (%)^a^		Protection (%)^a^	
PfCSP	37.5****	1.47***	33.3**	0.48*
PfTRAP	0	−0.06	30*	0.03
PfLSAP1	0	0.05	30	0
PfETRAMP5	0	0.12	10	0
PfCelTOS	0	−0.25*	0	0.28
PfUIS3	12.5***	1.42**	0	1.1
PfLSAP2	87.5****	1.09	70***	0.29
PfFalstatin	0	0.59**	10****	0.97****
PfLSA1	87.5****	2.13	87.5****	2
PfLSA3^c^	12.5*	0.35*	0	0.22

^a^Percentage of vaccinated mice that were sterilely protected from challenge with 1000 chimeric sporozoites i.v., n = 8–10.

^b^The median delay (days) in time to 1% parasitaemia, calculated by: (time to 1% of vaccinee) – (average time to 1% of naïve controls). The difference in survival was generated using Kaplan-Meier survival curves with statistical significance assessed using the Log-Rank (Mantel-Cox) Test, *p < 0.05-0.01 **p < 0.01-0.001 ***p < 0.001 ****p < 0.0001.

^c^For PfLSA3 challenge, the chimeric sporozoite dose was increased to 2000 sporozoites per mouse in order to infect all naïve controls.
