# Supplementary material for: Comparative assessment of vaccine vectors encoding ten malaria antigens identifies two protective liver-stage candidates
Source: Sci Rep. 2015 Jul 3;5:11820. doi: 10.1038/srep11820 (PMC4490344; doi:10.1038/srep11820)
Supplement: Supplementary Information [file srep11820-s1.pdf]

Supplementary Information

Comparative assessment of vaccine vectors encoding ten malaria antigens identifies two protective liver-stage candidates

Rhea J. Longley[1,¶,‡,*], Ahmed M. Salman[1,2,¶], Matthew G. Cottingham[1], Katie Ewer[1], Chris J. Janse[2], Shahid M. Khan[2], Alexandra J. Spencer[1,†], Adrian V. S. Hill[1,†]

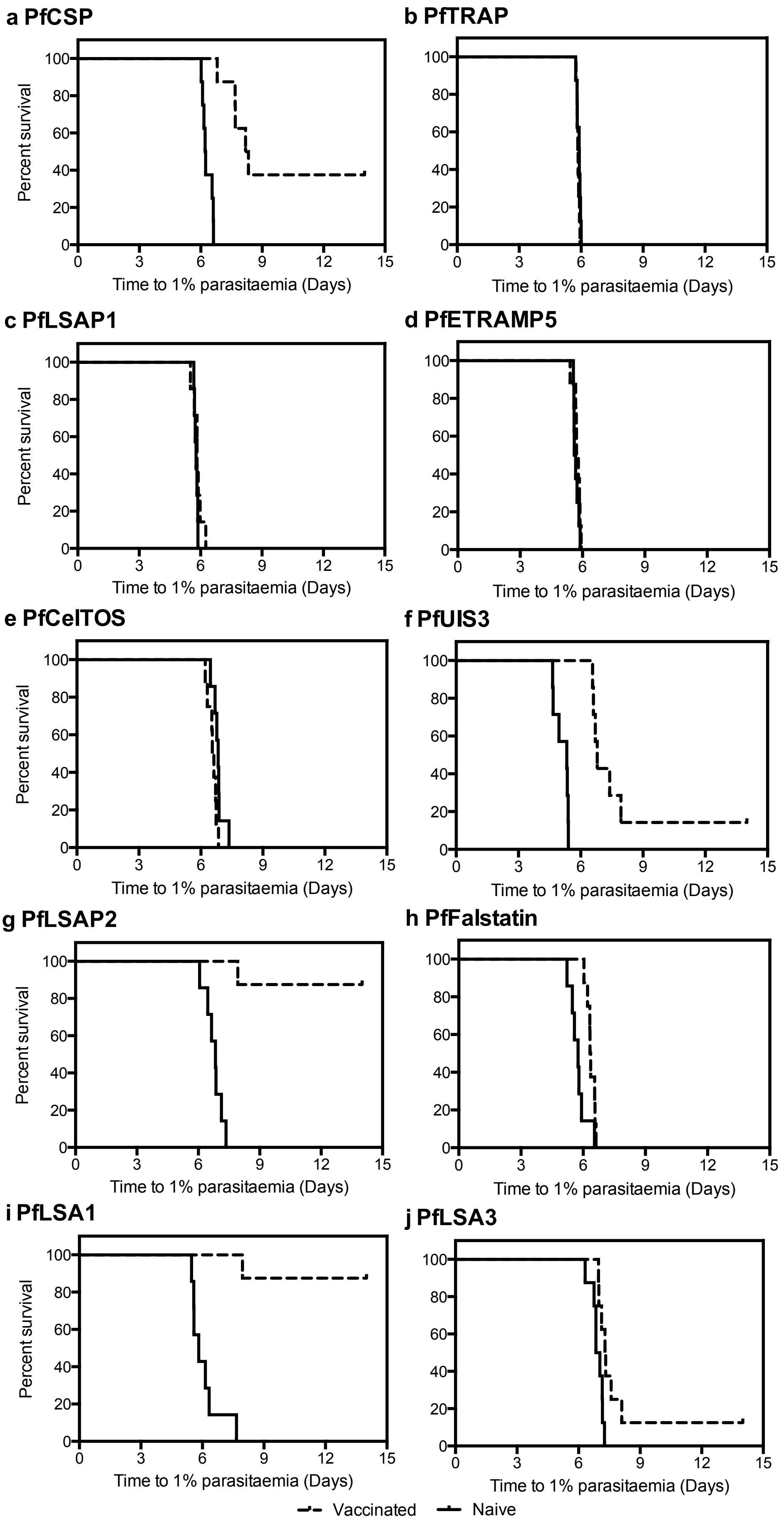
a PfCSP
b PfTRAP
c PfLSAP1
d PfETRAMP5
e PfCelTOS
f PfUIS3
g PfLSAP2
h PfFalstatin
i PfLSA1
j PfLSA3
Percent survival
0
20
40
60
80
100
Time to 1% parasitaemia (Days)
0
3
6
9
12
15
Vaccinated
Naive

Figure S1. ChAd63-MVA *P. falciparum* vaccines can provide protection in BALB/c mice. (a-j) Eight vaccinated and eight naïve mice were challenged with 1000 chimeric sporozoites i.v. The Kaplan-Meier curves illustrate the time to 1% parasitaemia, whilst statistical significance between the survival curves was assessed using the Log-Rank (Mantel-Cox) Test, (**a**) PfCSP p=0.03, (**b**) PfTRAP p=0.3, (**c**) PfLSAP1 p=0.2, (**d**) PfETRAMP5 p=0.3, (**e**) PfCelTOS p=0.03, (**f**) PfUIS3 p=0.0001, (**g**) PfLSAP2 p<0.0001, (**h**) PfFalstatin p=0.007, (**i**) PfLSA1 p<0.0001 and (**j**) PfLSA3 p=0.01. For the PfLSA3 challenge, the chimeric sporozoite dose was increased to 2000 sporozoites per mouse in order to infect all naïve controls.

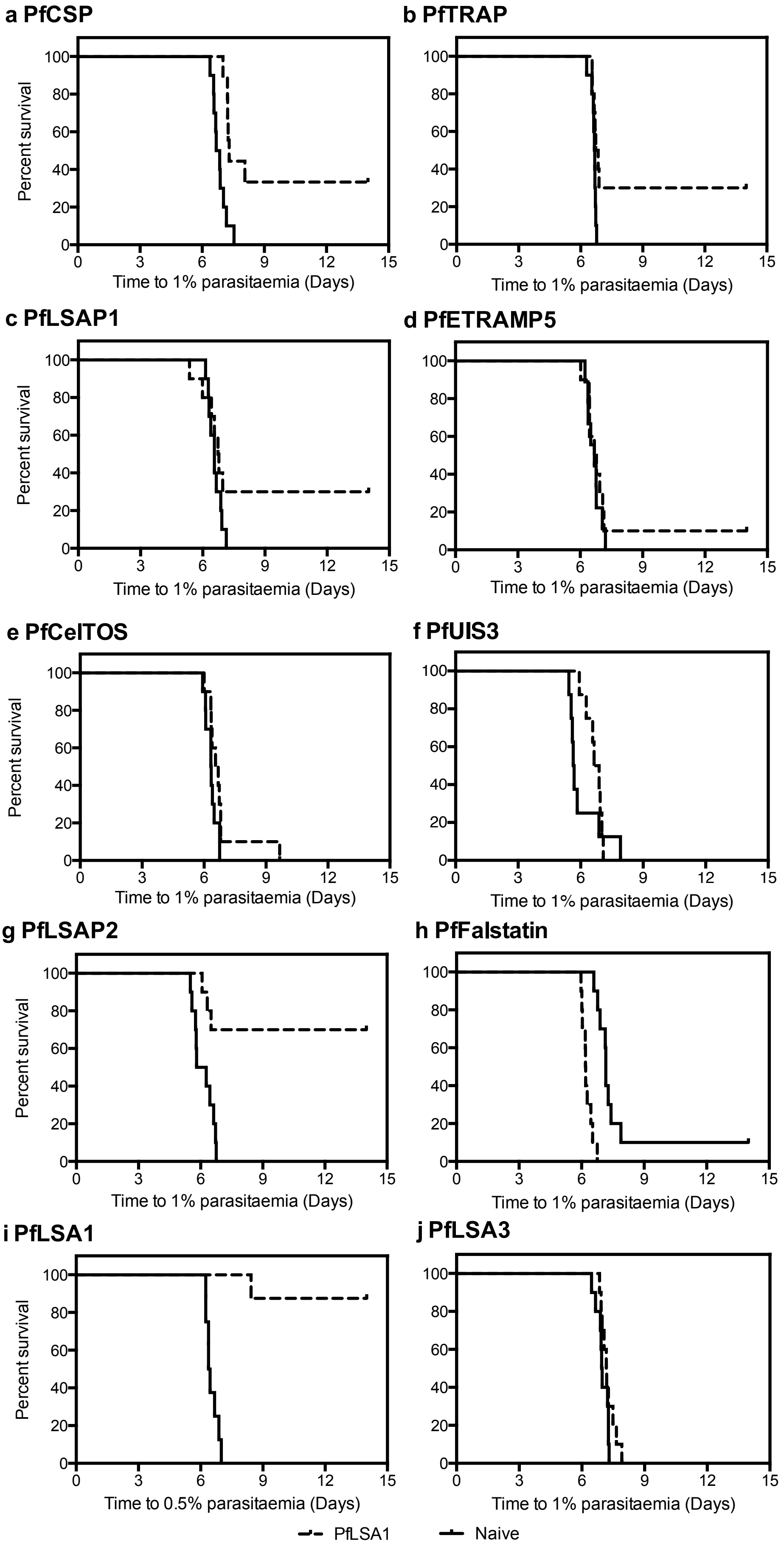
a PfCSP
b PfTRAP
c PfLSAP1
d PfETRAMP5
e PfCelTOS
f PfUIS3
g PfLSAP2
h PfFalstatin
i PfLSA1
j PfLSA3
Percent survival
Time to 1% parasitaemia (Days)
Time to 0.5% parasitaemia (Days)
0
20
40
60
80
100
0
3
6
9
12
15
PfLSA1
Naive

Figure S2. ChAd63-MVA *P. falciparum* vaccines can provide protection in CD-1 mice. (**a-j**) Eight to ten vaccinated and eight to ten naïve mice were challenged with 1000 chimeric sporozoites i.v. The Kaplan-Meier curves illustrate the time to 1% parasitaemia, whilst statistical significance between the survival curves was assessed using the Log-Rank (Mantel-Cox) Test: (**a**) PfCSP p=0.001, (**b**) PfTRAP p=0.02, (**c**) PfLSAP1 p=0.16, (**d**) PfETRAMP5 p=0.45, (**e**) PfCelTOS p=0.097, (**f**) PfUIS3 p=0.25, (**g**) PfLSAP2 p=0.0009 (**h**) PfFalstatin p<0.0001, (**i**) PfLSA1 p<0.0001 and (**h**) PfLSA3 p=0.15. For the PfLSA3 challenge, the chimeric sporozoite dose was increased to 2000 sporozoites per mouse in order to infect all naïve controls.

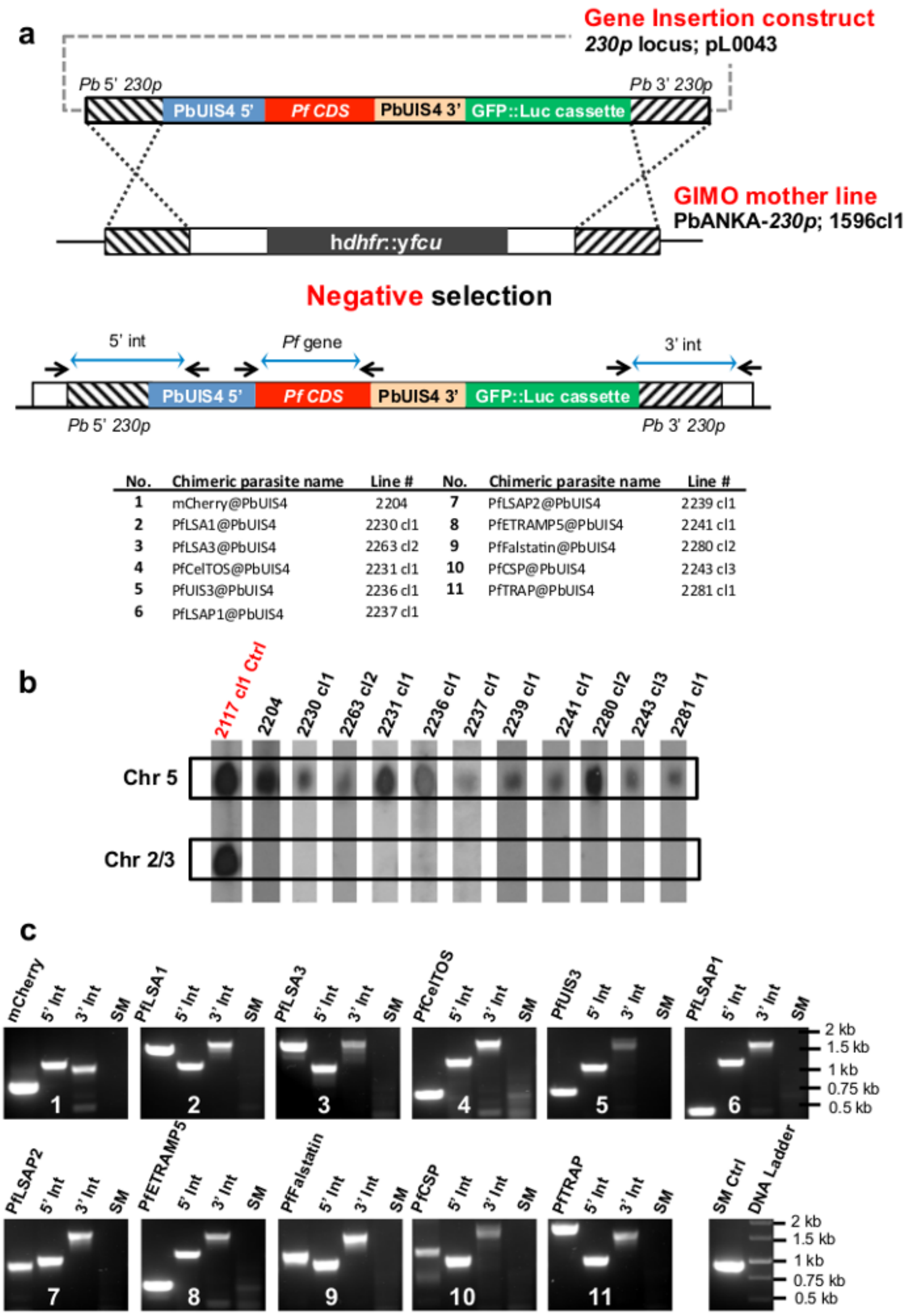

No.	Chimeric parasite name	Line #	No.	Chimeric parasite name	Line #
1	mCherry@PbUIS4	2204	7	PfLSAP2@PbUIS4	2239 cl1
2	PfLSA1@PbUIS4	2230 cl1	8	PfETRAMP5@PbUIS4	2241 cl1
3	PfLSA3@PbUIS4	2263 cl2	9	PfFalstatin@PbUIS4	2280 cl2
4	PfCelTOS@PbUIS4	2231 cl1	10	PfCSP@PbUIS4	2243 cl3
5	PfUIS3@PbUIS4	2236 cl1	11	PfTRAP@PbUIS4	2281 cl1
6	PfLSAP1@PbUIS4	2237 cl1			

Figure S3. All chimeric *P. berghei* parasite clones contain the correct genotype. (**a**) Schematic representation showing the introduction of the constructs that contain the '*P. falciparum* antigen expression-cassette' into the *230p* locus of the *P. berghei*

ANKA GIMO mother line by GIMO-transfection using negative selection (5-FC). Black arrows: location of PCR primers used for diagnostic PCR-analysis (see panel **c**). CDS, coding sequence. (**b**) Southern analysis of chromosomes (Chr) of chimeric parasite lines separated by pulsed-field gel electrophoresis to confirm integration of the DNA construct in the GIMO locus (*230p* on Chr 3), shown as the removal of the h*dhfr::*y*fcu* SM cassette in cloned chimeric parasites compared to a control probe recognising Chr 5. As an additional control (Ctrl), parasite line 2117cl1 is also shown as it retains h*dhfr::*y*fcu* SM in the *230p* locus on Chr 3. (**c**) Diagnostic PCR analysis of chimeric parasite lines confirming correct integration of the *P. falciparum* antigen expression cassettes. Correct integration in all lines is shown by the absence of the h*dhfr*::y*fcu* SM, the presence of the *P. falciparum* gene coding sequence and the correct integration of the construct into the genome at both the 5'and 3'regions (5' int and 3' int).

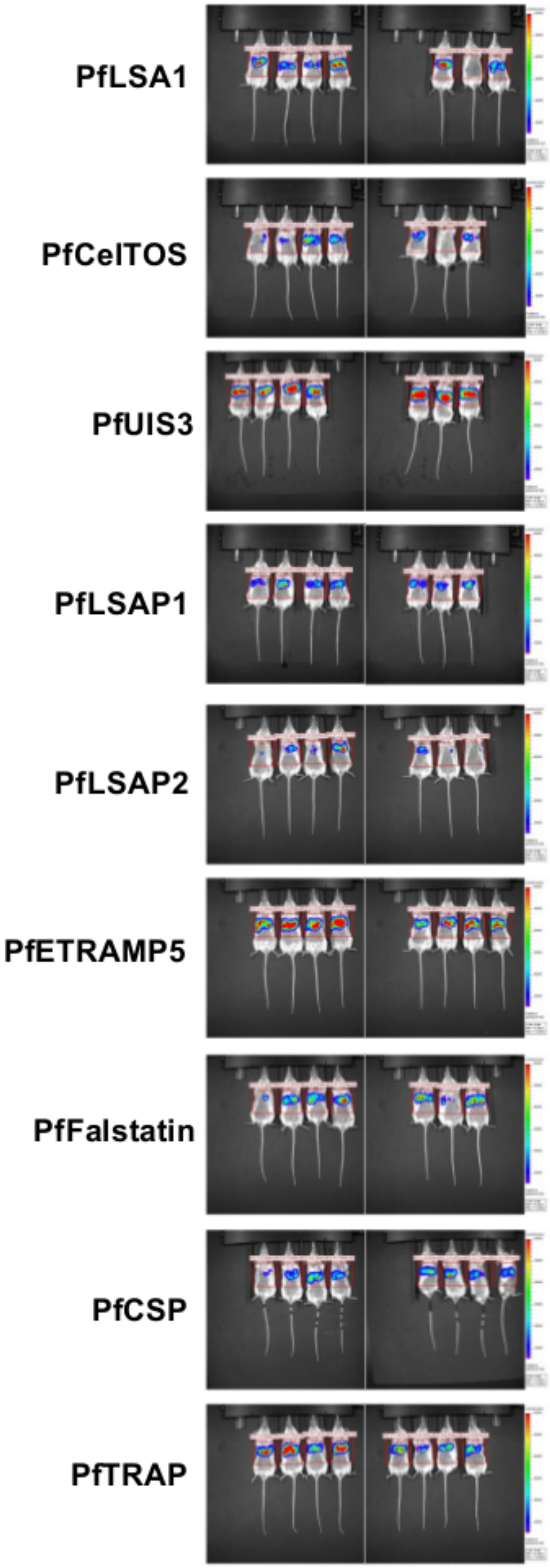
PfLSA1
PfCelTOS
PfUIS3
PfLSAP1
PfLSAP2
PfETRAMP5
PfFalstatin
PfCSP
PfTRAP

Figure S4. All chimeric *P. berghei* parasites express the inserted antigen at the liver-stage of infection. As each chimeric parasite also contained a GFP::luciferase reporter cassette, luciferase expression in chimeric *P. berghei* parasites was measured at 44 hours post-injection of 1000 sporozoites i.v. into seven to eight BALB/c mice, using the IVIS 200 *in vivo* imager. All chimeric parasites expressed luciferase at the liver-stage, providing an indirect confirmation of antigen expression at the liver-stage.

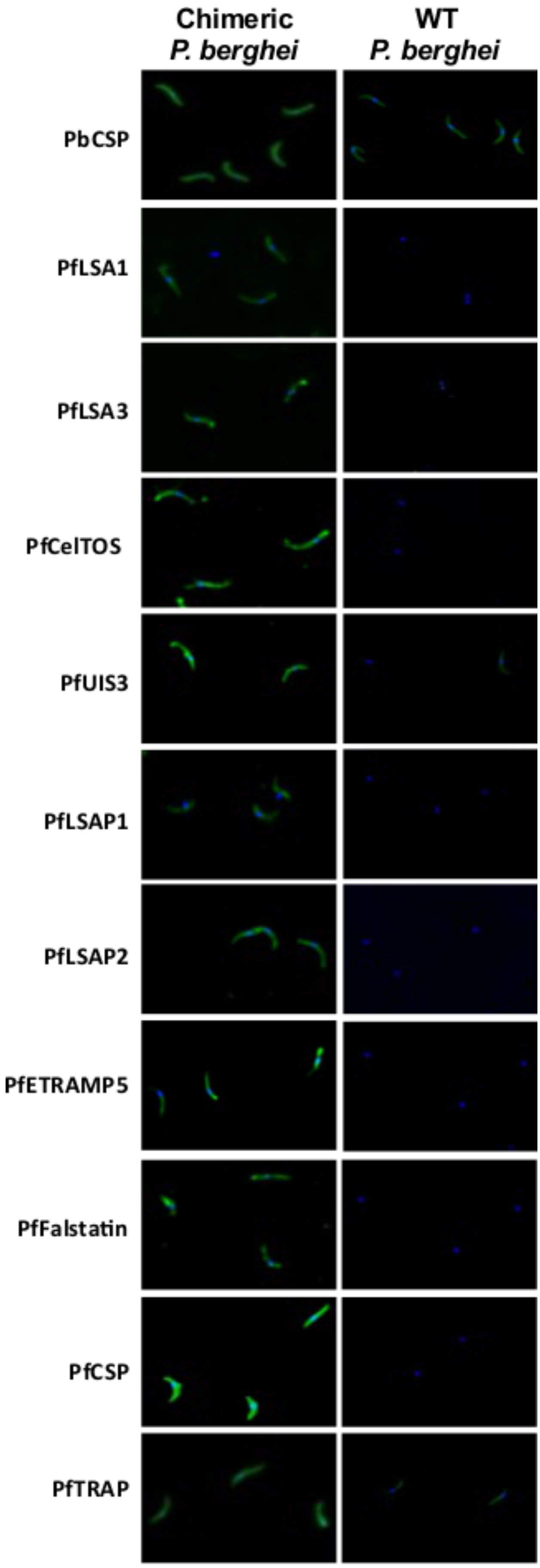
Chimeric
P. berghei
WT
P. berghei
PbCSP
PfLSA1
PfLSA3
PfCelTOS
PfUIS3
PfLSAP1
PfLSAP2
PfETRAMP5
PfFalstatin
PfCSP
PfTRAP

Figure S5. All chimeric *P. berghei* sporozoites express the inserted *P. falciparum* antigen. Chimeric salivary-gland sporozoites were stained with sera from vaccinated mice or with monoclonal antibodies where available (PbCSP 3D11 and PfCSP 2A10), with green fluorescence indicating the presence of the protein (Alexa Fluor 488). As a control, wild-type (WT) *P. berghei* sporozoites were stained with the same antibodies or sera.

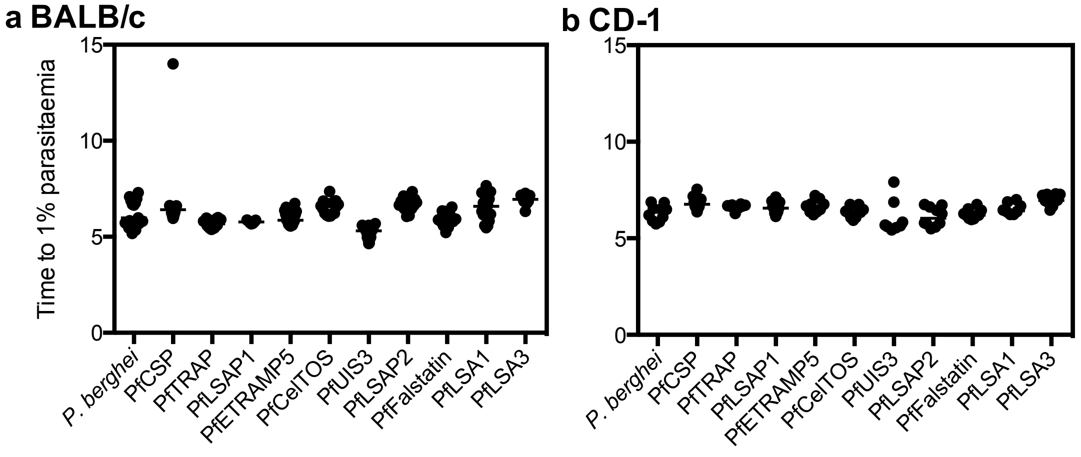

Figure S6. Infectivity of the chimeric parasites in mice compared to wild-type *P. berghei.* Time to 1% parasitaemia of the chimeric parasites compared to wild-type parasites following injection of 1000 sporozoites i.v, into (**a**) 7-22 BALB/c and (**b**) 8-10 CD-1 mice. Both median and individual data points are shown. All chimeric parasites are statistically comparable to wild-type (Mann-Whitney test), except for PfUIS3, $p<0.0001$, and PfLSA3, $p=0.02$, both in BALB/c, and PfCSP, $p=0.009$, and PfLSA3, $p=0.0004$, in CD-1 mice.

Table S1. Vaccine construct details.

Antigen	Gene ID[a]	Size[b]	Predicted Structure	tPA	Final size[b]
PfLSAP1	PF3D7_1201300	318	Signal peptide, two transmembrane domains	No	347
PfETRAMP5	PF3D7_0532100	543	Signal peptide, transmembrane domain, repetitive region	Yes	662
PfCelTOS	PF3D7_1216600	546	Signal peptide	Yes	665
PfUIS3	PF3D7_1302200	687	Signal peptide, two transmembrane domains	No	716
PfLSAP2	PF3D7_0202100	906	Non-secretory, transmembrane domain	Yes	1025
PfFalstatin	PF3D7_0911900	1239	Signal peptide	Yes	1358
PfLSA1	PF3D7_1036400	3486	Signal peptide, repetitive regions	Yes	1502
PfLSA3	PF3D7_0220000	4674	Non-secretory, two transmembrane domains, repetitive regions	No	4259

[a] PlasmoDB Gene ID. [b] Size in base pairs.
